# Role of PAI-1 in hepatic steatosis and dyslipidemia

**DOI:** 10.1038/s41598-020-79948-x

**Published:** 2021-01-11

**Authors:** Joshua A. Levine, Carlota Oleaga, Mesut Eren, Ansel P. Amaral, Meng Shang, Elizabeth Lux, Sadiya S. Khan, Sanjiv J. Shah, Yasuhiro Omura, Nathalie Pamir, Joshua Hay, Grant Barish, Toshio Miyata, Hagai Tavori, Sergio Fazio, Douglas E. Vaughan

**Affiliations:** 1 Department of Medicine, Northwestern University Feinberg School of Medicine, Arkes Pavilion, Suite 2330, 676 N. St. Clair Street, Chicago, IL 60611-2927 USA; 2 Center for Preventive Cardiology, Knight Cardiovascular Institute, Oregon Health and Science University, Portland, OR USA; 3 Department of Medicine, Jesse Brown VA Medical Center, Chicago, IL USA; 4 Department of Molecular Medicine and Therapy, United Centers for Advanced Research and Translational Medicine, Tohoku University Graduate School of Medicine, Miyagi, Japan

**Keywords:** Metabolic syndrome, Dyslipidaemias, Non-alcoholic fatty liver disease

## Abstract

Plasminogen activator inhibitor 1 (PAI-1) is a functional biomarker of the metabolic syndrome. Previous studies have demonstrated that PAI-1 is a mechanistic contributor to several elements of the syndrome, including obesity, hypertension and insulin resistance. Here we show that PAI-1 is also a critical regulator of hepatic lipid metabolism. RNA sequencing revealed that PAI-1 directly regulates the transcriptional expression of numerous genes involved in mammalian lipid homeostasis, including PCSK9 and FGF21. Pharmacologic or genetic reductions in plasma PAI-1 activity ameliorates hyperlipidemia in vivo. These experimental findings are complemented with the observation that genetic deficiency of PAI-1 is associated with reduced plasma PCSK9 levels in humans. Taken together, our findings identify PAI-1 as a novel contributor to mammalian lipid metabolism and provides a fundamental mechanistic insight into the pathogenesis of one of the most pervasive medical problems worldwide.

## Introduction

Obesity-associated metabolic syndrome, which affects a growing percentage of the American population, increases the risk of cardiovascular disease (CVD) and related mortality^1–3^. The diagnosis of metabolic syndrome is based on the presence of central obesity, hypertension, dyslipidemia, and insulin resistance^4^. Other clinical features and biomarkers have been linked to obesity and metabolic syndrome, including increased plasma levels of the serine protease inhibitor plasminogen activator inhibitor-1 (PAI-1)^5,6^.

In mammals, PAI-1 serves as the primary rapid acting inhibitor of tissue-type plasminogen activator (t-PA) and urokinase-type plasminogen activator (uPA). Numerous studies have identified strong statistical correlations between plasma levels of PAI-1 and each of the individual components of the metabolic syndrome and these relationships are additive, with more components yielding higher PAI-1 levels^7,8^. Plasma PAI-1 levels strongly correlate with body mass index (BMI)^7^, and effectively predict the development of the metabolic syndrome and type 2 diabetes mellitus (T2DM)^8–12^. Similar to humans, plasma PAI-1 levels are substantially increased in murine models of obesity and T2DM^13,14^. Mice with genetic deficiency of PAI-1 are resistant to diet-induced obesity, hepatic steatosis, and insulin resistance^13,15^. Furthermore, pharmacologic inhibition of PAI-1 with an orally active small molecule inhibitor preserves insulin sensitivity and prevents hepatic steatosis in mice^16–18^. These links between PAI-1 and insulin sensitivity have recently been confirmed in humans. A rare loss-of-function mutation in the gene that codes for PAI-1 (*SERPINE*1) has been identified in a kindred of Old-Order Amish in northeastern Indiana. Heterozygous carriers of the mutation have lower fasting insulin levels and are protected from the development of T2DM relative to unaffected members of the same kindred^19^.

Despite the strong evidence suggesting that PAI-1 plays a key role in obesity-related T2DM and NAFLD, no studies have described or investigated a mechanistic link between PAI-1 and dyslipidemia. In this study, we show that PAI-1 broadly regulates the transcription of numerous components of the lipid metabolic machinery in the liver. This newly discovered mechanistic relationship allows for the identification of PAI-1 as a singular factor that directly contributes to each of the clinical components of the metabolic syndrome.

## Results

### PAI-1 inhibition reduces PCSK9 expression

**Figure 1:**
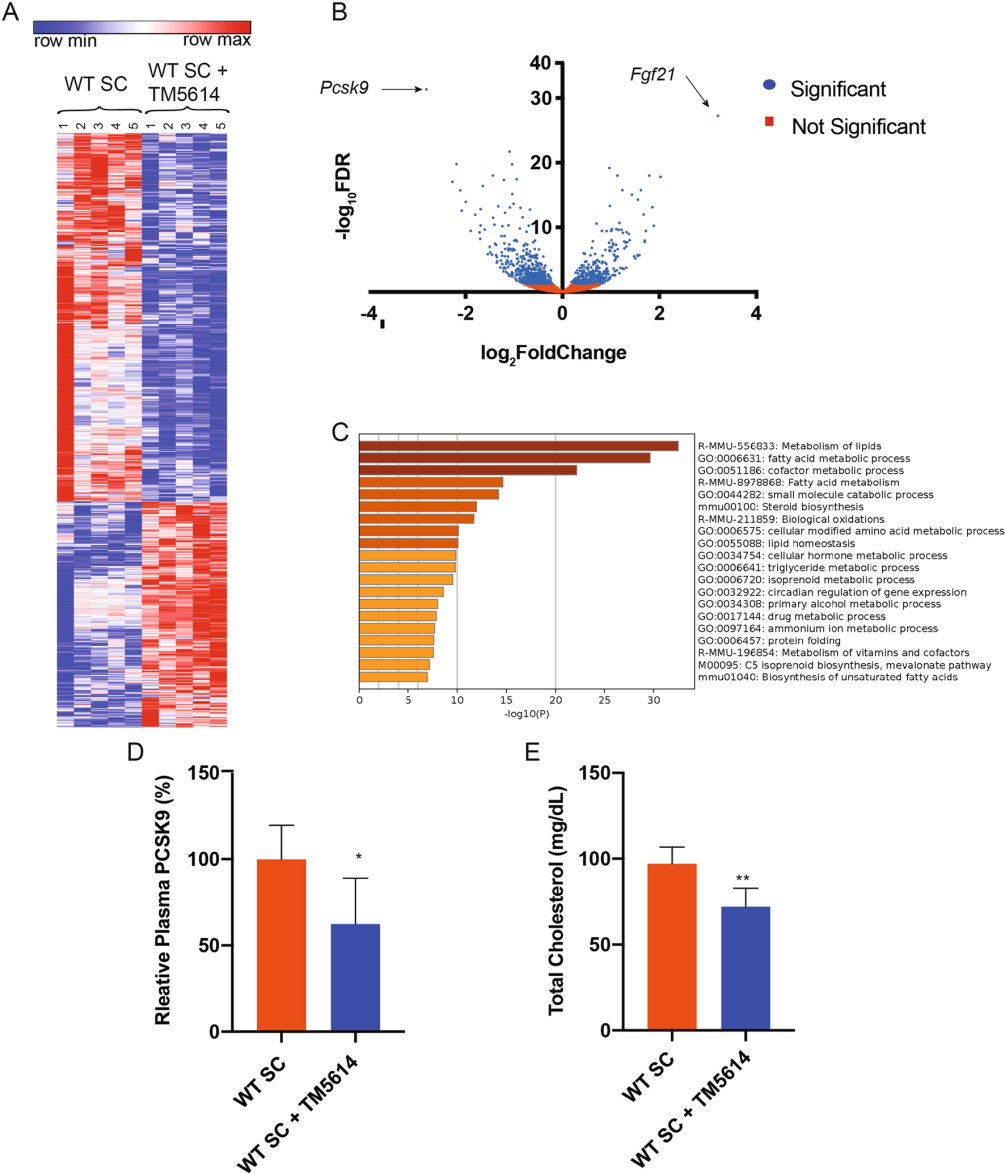
Pharmacological inhibition of PAI-1 with TM5614 regulates *Pcsk9*. 20 week-old wild-type C57BL/6 J male mice fed SC were administered a PAI-1 inhibitor, TM5614, given orally (20 mg/kg/day) for 10 days. At the endpoint hepatic mRNA and murine plasma were analyzed. (**A**) Hierarchical clustering of expression changes seen by RNA-seq. Heat map created using Morpheus (https://software.broadinstitute.org/morpheus/). (**B**) Volcano plot representation of all gene expression changes. Blue points represent statistically significant changes and red points represent non-significant changes. (**C**) Gene ontology analysis of statistical differentially expressed genes. Data generated using Metascape (https://metascape.org/gp/index.html#/main/step1). (**D**) Murine plasma PCSK9 levels. (**E**) Total Cholesterol levels in mouse plasma. Values are expressed as mean ± SEM (n = 5), * p < 0.05, ** p < 0.01.

Selective pharmacologic inhibition of PAI-1 prevents the development of NAFLD in mouse models of diet-induced obesity^16,17^. To investigate the mechanistic role of PAI-1 in hepatic lipid metabolism, RNA sequencing (RNA-seq) was performed on hepatic mRNA from mice treated with TM5614, a novel orally active small molecule inhibitor of PAI-1, and compared their gene expression patterns with those from hepatic mRNA collected from age-matched littermate control mice consuming a standard chow (SC) diet. Cluster analysis indicated homogeneity amongst samples in each group, with numerous changes induced by blocking PAI-1 in RNA expression across the genome (Fig. 1A). Hierarchical clustering and gene ontology analysis revealed that short-term administration of a PAI-1 inhibitor generated a coordinated, robust, and significant alteration in the expression of numerous genes involved in metabolism of lipids (160 genes) and fatty acid metabolism (156 genes) in response to treatment (Fig. 1C, S1A). Differential gene expression analysis indicated that *Proprotein convertase subtilisin/kexin type 9* (*Pcsk9*) was the most highly downregulated gene transcript in mice treated with TM5614 (86% decrease, adjusted* p* = 4.34 × 10^−32^, Fig. 1B), whereas *Fibroblast growth factor 21* (*FGF21*) was the most upregulated transcript (568% increase, adjusted * p* = 5.66 × 10^−28^, Fig. 1B). Consistent with these transcriptional changes, treatment with TM5614 led to a 40% decrease in plasma PCSK9 levels (Fig. 1D, * p* < 0.05) and this was accompanied by a 25% reduction in plasma cholesterol levels (Fig. 1E, * p* < 0.01). In humans, circulating PCSK9 reduces low density lipoprotein receptor (LDLR) levels by targeting the receptor for degradation, thus impairing LDL cholesterol (LDL-C) clearance from circulation and increasing plasma LDL-C levels^20^. Wild-type (WT) mice have minimal levels of LDL-C, as their plasma cholesterol is mostly carried by high density lipoproteins (HDL)^21^. In contrast to humans, both LDL and HDL particles in mice can be cleared by the LDLR^22^. As expected, the decrease in total cholesterol caused by PAI-1 inhibition was driven by a reduction in HDL cholesterol (HDL-C) level (Figure S2A,B). Consistent with these findings in vivo, incubation with TM5614 in HepG2 cells resulted in a reduction in PCSK9 protein levels (48% intracellularly (* p* < 0.05), 69% extracellularly (* p* < 0.01) and a concomitant increase in LDLR protein levels (65%, * p* < 0.001), suggesting that PAI-1 regulation of PCSK9 levels has an impact on cholesterol clearance mediated by increased LDLR levels (Figure S3).

**Figure 2:**
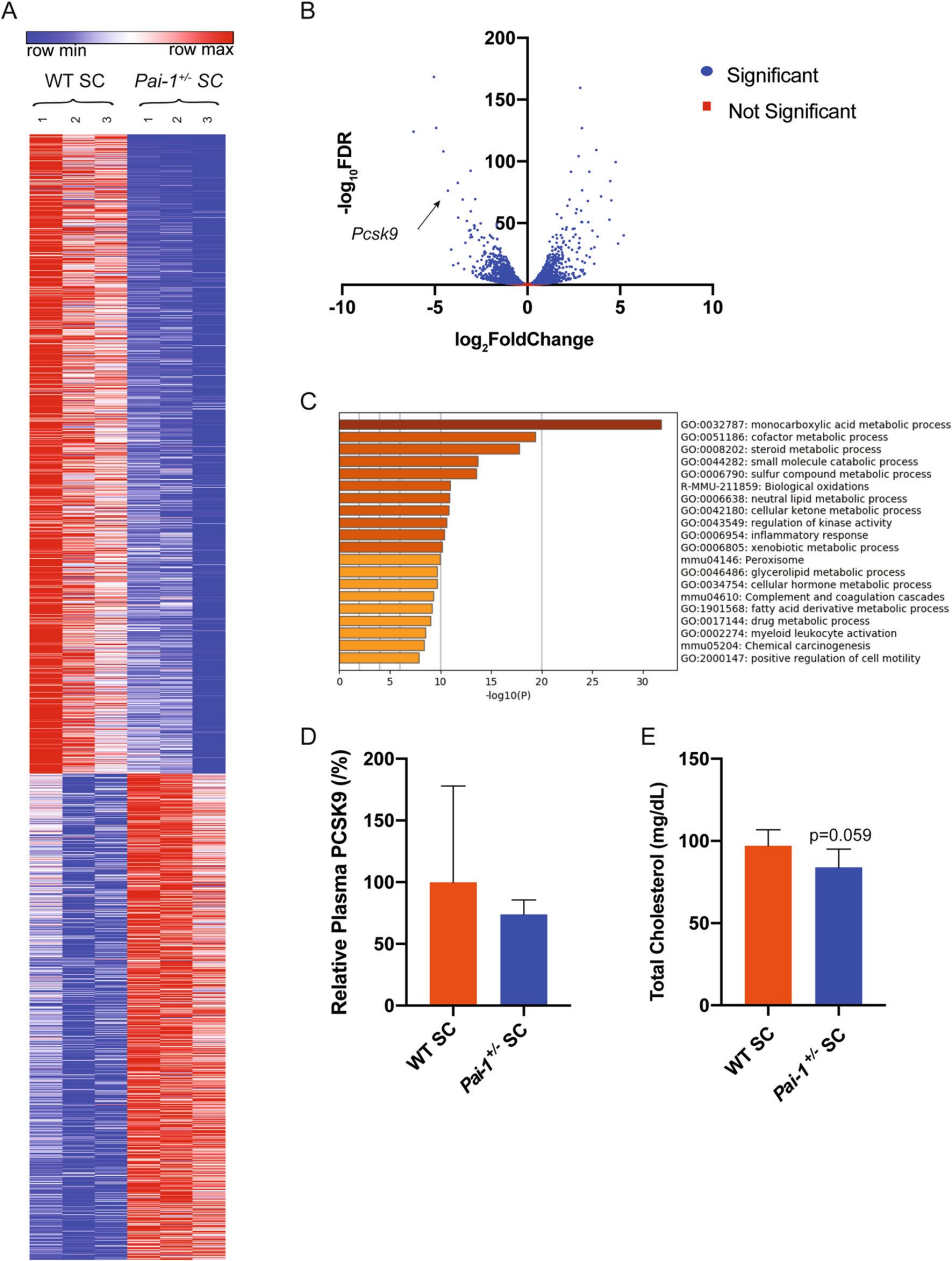
Lipid metabolism is differentially regulated in PAI-1 heterozygosity. RNA-SEQ was performed on hepatic mRNA from 20 week-old *Pai-1*^+/−^ and littermate WT control C57BL/6 J male mice fed SC (n = 3). (**A**) Hierarchical clustering of expression changes seen by RNA-seq. Heat map created using Morpheus (https://software.broadinstitute.org/morpheus/). (**B**) Volcano plot representation of all gene expression changes. Blue points represent statistically significant changes and red points represent non-significant changes. (**C**) Gene ontology analysis of statistical differentially expressed genes. Data generated using Metascape (https://metascape.org/gp/index.html#/main/step1). (**D**) Murine PCSK9 plasma levels. (**E**) Total cholesterol plasma levels. Values are express as mean ± SEM (n = 7).

To exclude the influence of an off-target effect of the drug on the observed alterations in hepatic gene expression, a similar set of studies was performed using heterozygous PAI-1 deficient mice (*Pai-1*^+/−^) and wild-type (WT) littermate controls. RNA-seq was performed on hepatic mRNA from 20 week-old mice maintained on the SC diet. Cluster analysis indicated homogeneity amongst samples in each group, with multiple changes in RNA expression across the genome induced by lower PAI-1 levels (Fig. 2A). Similar to the effects seen with the administration of the PAI-1 inhibitor, differential gene expression analysis identified *Pcsk9* as one the most highly downregulated gene transcript identified in *Pai-1*^+/−^ mice (Fig. 2B). Furthermore, *Pai-1* heterozygosity also resulted in a coordinated and robust alteration in the expression of genes involved in lipid metabolism (Figure S1B), and these results were corroborated by a gene ontology analysis (Fig. 2C). Plasma PCKS9 levels trended lower in *Pai-1*^+/−^ mice but did not reach statistical significance (Fig. 2D). Pharmacological inhibition of PAI-1 shared 771 differentially expressed genes with heterozygous *Pai-1* deficiency (Figure S1C). The observed 16% reduction in decrease in total cholesterol levels (Fig. 2E; * p* = 0.059) suggest that the observed transcriptional changes generate the expected changes in lipid homeostasis.

### PAI-1 inhibition in hypercholesterolemia lowers PCSK9 levels and corrects dyslipidemia in vivo

**Figure 3:**
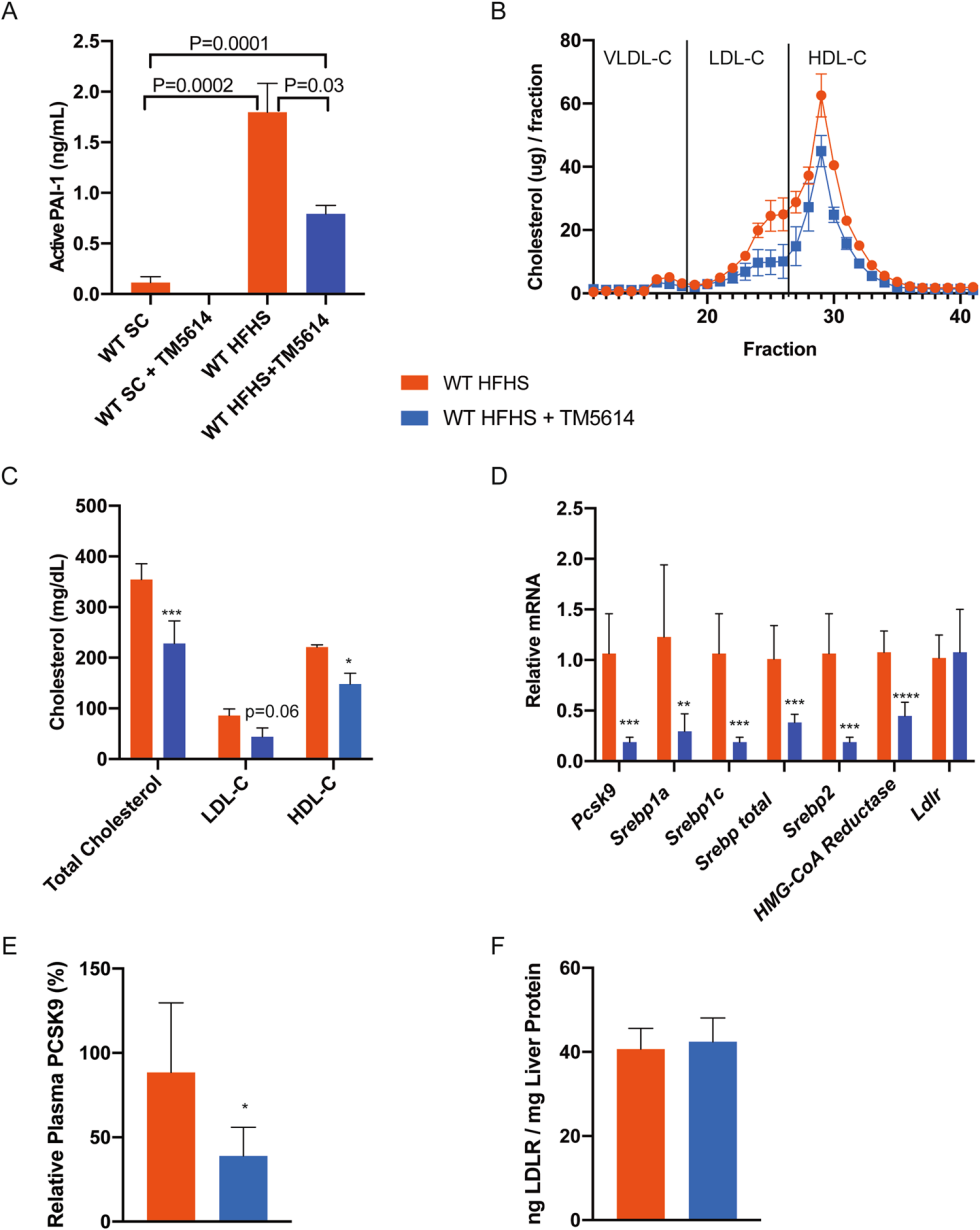
Long-term TM5614 treatment leads to a reduction in circulatory cholesterol and PCSK9 in mice fed a HFHS diet. WT C57BL/6 J male mice fed HFHS diet with and without TM5614. (**A**) Active PAI-1 levels (ng/mL). (**B**) Cholesterol distribution on plasma lipoprotein fractions resolved by FPLC from pooled samples (n = 2 for HFHS, n = 3 for HFHS + TM5614) (2-way ANOVA * p* = 0.03). (**C**) Quantification of cholesterol in each lipoprotein fraction calculated using area under the curve from the FPLC profile. (**D**) Hepatic mRNA expression of cholesterol regulatory pathway nodes. (**E**) Plasma PCSK9 levels (ng/mL). (**F**) LDLR protein levels from liver lysates (ng LDLR/mg Liver Protein). Values are express as mean ± SEM (n = 7), * p < 0.05, ** p < 0.01, *** p < 0.001.

To investigate further the effects of PAI-1 inhibition on cholesterol metabolism, WT C57BL/6 J mice were fed a high-fat, high-sugar (HFHS) diet to induce obesity and hyperlipidemia. Mice were placed on HFHS diet for 16 weeks, and then treatment with TM5614 was initiated in half of the experimental animal cohort for an additional 10 weeks, while the other half of the cohort remained, untreated, on the HFHS diet. The HFHS diet increased total cholesterol, LDL-C, and HDL-C, as expected. There were no differences between baseline or end-of-study weight between groups (Figure S4A,B). Consistent with prior studies, inhibition with TM5614 was associated with preserved glucose tolerance and decreased hepatic steatosis (Figure S4C–E). The HFHS diet produced a 16-fold increase in plasma levels of active PAI-1 (* p* < 0.001), whereas TM5614 administration reduced plasma levels of active PAI-1 by 56% (* p* < 0.05) (Fig. 3A). Inhibition of PAI-1 led to a lowering of plasma total cholesterol by 37% (* p* < 0.05), LDL-C by 48% (* p* = 0.06), and HDL-C by 33% (* p* < 0.05) (Fig. 3B,C). Hepatic *Pcsk9* mRNA expression was reduced by 81% (* p* < 0.001) in mice fed a HFHS supplemented with TM5614. Drug treatment also reduced the expression of other genes involved in lipid metabolism (Fig. 3D), including *Srebp1a* (70%, * p* < 0.01), *Srebp1c* (81%, p  < 0.001), *Srebp2* (42%, * p*  < 0.001), and *HMG-CoA reductase* (55%, * p* < 0.0001). The reduction in *Pcsk9* mRNA expression was associated with a 55% reduction in the corresponding plasma levels of PCSK9 protein (* p* < 0.01, Fig. 3E), while corresponding measures of *Ldlr* mRNA and protein were essentially unchanged (Figs. 3D,F).

**Figure 4:**
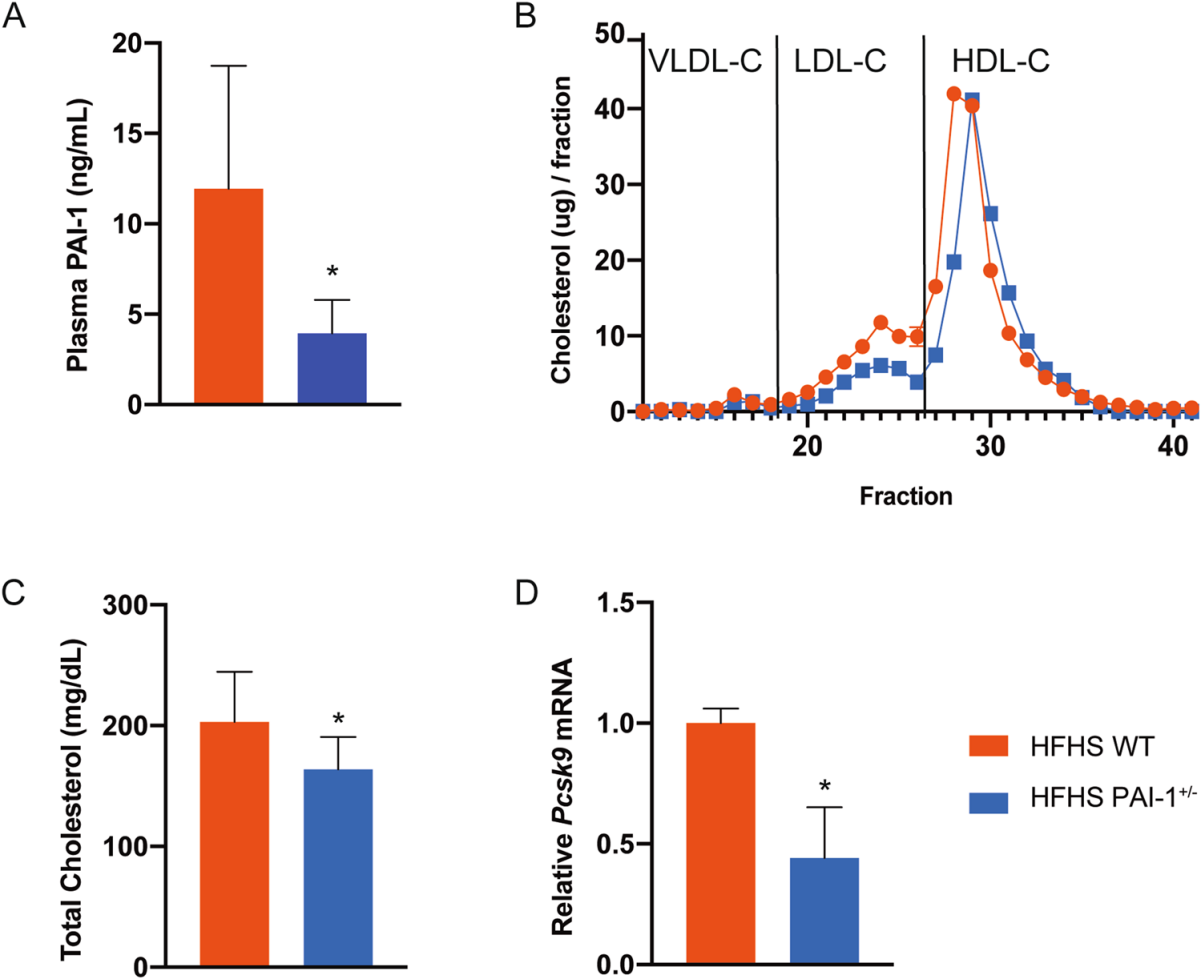
PAI-1 heterozygosity is associated with lower *Pcsk9* and cholesterol profiles in HFHS fed mice. 20 week-old wild-type or *Pai-1*^+/−^ C57BL/6 J male mice fed HFHS diet. At the endpoint murine hepatic mRNA and plasma were analyzed. (**A**) Plasma PAI-1 levels. (**B**) Cholesterol content profile of the lipoprotein fractions resolved by FPLC from pooled samples (n = 6 for WT, n = 7 for *Pai-1*^+/−^). (**C**) Plasma total cholesterol. (**D**) Hepatic PCSK9 mRNA expression normalized to GAPDH. Values are expressed as mean ± SEM (n = 6–7), * p < 0.05.

We designed and performed similar studies using heterozygous null mice with partial PAI-1 deficiency (*Pai-1*^+/−^) fed a HFHS diet for 16 weeks. As expected, plasma PAI-1 levels in these mice are approximately 50% (* p* < 0.05) of those of littermate controls (Fig. 4A). In these studies, partial PAI-1 deficiency was associated with a 20% lower plasma cholesterol level (* p* < 0.05), driven by a decrease in LDL-C and HDL-C (Fig. 4B,C). Hepatic *Pcsk9* transcript levels were reduced by 50% (* p* < 0.05) (Fig. 4D). Plasma levels of PCSK9 were also reduced by 61% (* p* < 0.001) (Figure S5). A comparison of PCSK9 plasma levels in all of the experimental groups described in this paper is shown in Figure S5. Taken together, these data show that PAI-1 directly influences the expression of PCSK9.

### Relationship between PAI-1 and PCSK9 in humans

**Figure 5:**
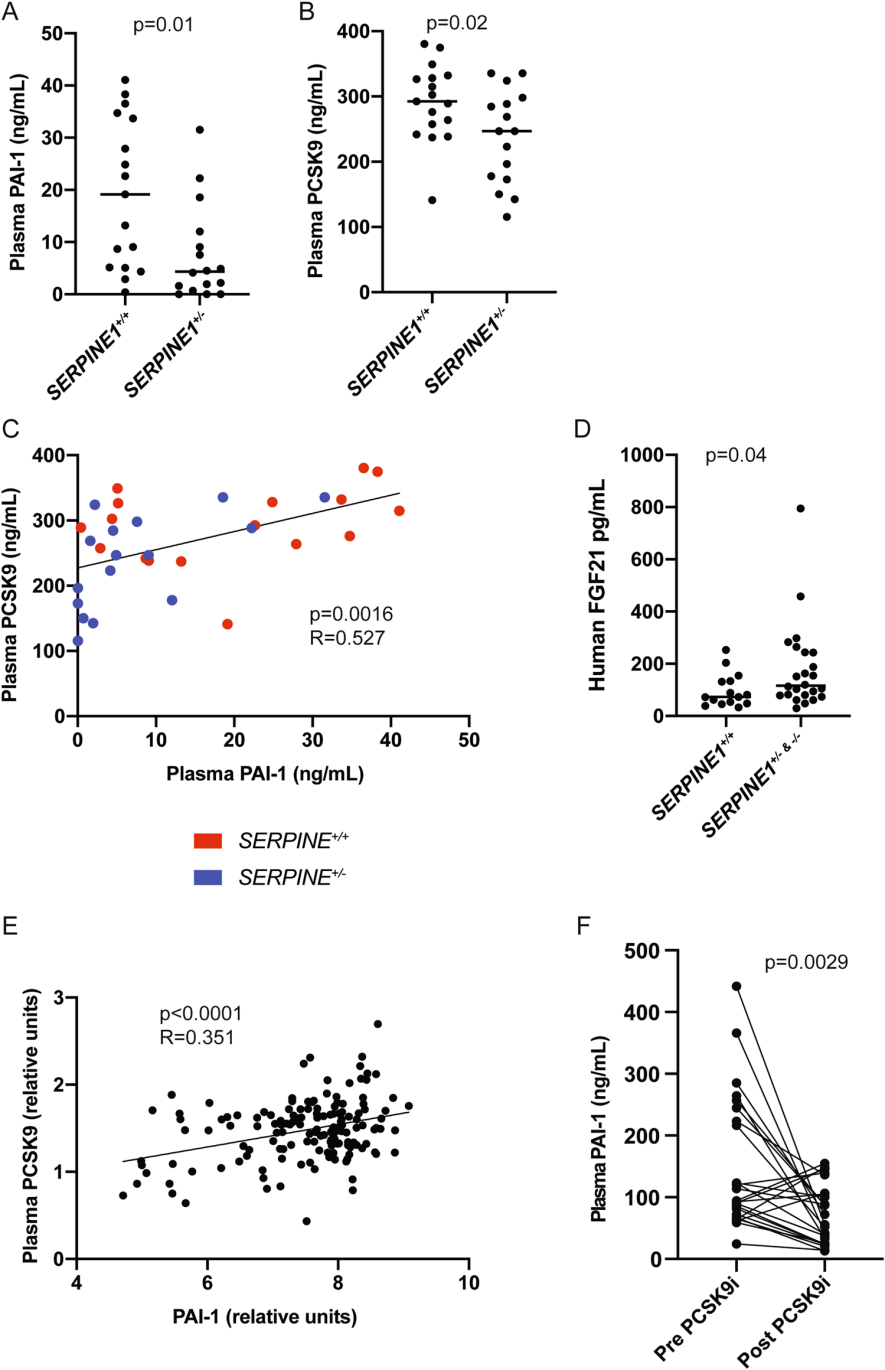
Plasma PCSK9 levels correlate with PAI-1 in humans. Characterization of the Amish cohort human plasma [(**A**) PAI-1 levels, (**B**) PCSK9 levels, (**C**) correlation between plasma PAI-1 vs. plasma PCSK9 levels, and (**D**) FGF21 levels] (values are express as mean ± SEM (n = 7 affected, 16 carriers*,* 17 unaffected). (**E**) Relationship between PCSK9 and PAI-1 levels in a separate cohort of HFpEF patients, values are express as mean ± SEM (n = 147). (**F**) PAI-1 levels in human plasma from a cohort of patients with hyperlipidemia before and during treatment with a PCSK9 inhibitor (values are express as mean ± SEM, n = 28).

Next, we sought to determine if genetic deficiency in *PAI-1* affects plasma PCSK9 levels in humans. We used frozen plasma samples previously obtained from members of the Indiana Swiss Amish community, which harbors a unique loss-of-function mutation in *SERPINE1*, the gene that codes for PAI-1^19^. As previously reported, plasma PAI-1 levels are reduced by 50% in heterozygous carriers (S*ERPINE1*^+/−^) of the genetic variant and are essentially undetectable in the rare homozygous (*SERPINE1*^−/−^) individuals (Fig. 5A, * p* < 0.001). Carriers of this mutation (n = 16, average age 45.1 ± 18.9 years) had significantly lower plasma PCSK9 levels compared to unaffected controls (n = 17, average age 42.0 ± 21.4 years) in the same community (Fig. 5B, * p* = 0.02). Furthermore, PCSK9 levels positively correlated with PAI-1 levels (Fig. 5C, r = 0.557, * p* < 0.001). In addition, carriers and affected individuals had significantly higher plasma levels of FGF21 (Fig. 5D).

To validate the relationship between plasma PAI-1 and PCSK9 levels in humans, we studied plasma samples from a second human cohort of adults (mean age 69.5, 66% female). This cohort included control patients and patients with heart failure and preserved ejection fraction (HFpEF). There was again a positive correlation between PAI-1 and PCSK9 levels in these individuals, similar to that seen in mice and in the Amish cohort (Fig. 5E, * p* < 0.0001; R = 0.351). Furthermore, we analyzed PAI-1 levels in hypercholesterolemic patients treated with therapeutic monoclonal antibodies that block PCSK9 binding to the LDLR (evolocumab and alirocumab). In aggregate, treatment with PCSK9 inhibitors was followed by a substantial increase in total plasma PCSK9 levels (Figure S6, * p* < 0.001) coupled with a significant reduction in plasma PAI-1 levels (Fig. 5F, * p* = 0.0029). These data suggest that modulation of PCSK9 activity has a novel and reciprocal effect on plasma PAI-1 levels.

## Discussion

In this study, we demonstrate for the first time that PAI-1 is directly involved in lipid metabolism. Previous clinical studies have reported that plasma PAI-1 levels correlate with levels of VLDL^23^. This physiological relationship was explained by direct effects of VLDL in inducing PAI-1 expression. We now demonstrate the presence of a converse operational paradigm, that pharmacologic or genetic reductions in PAI-1 alone are sufficient to produce alterations in lipid homeostasis. The administration of a specific PAI-1 inhibitor generates an unexpected and multifaceted hepatic transcriptional response in numerous genes involved in lipid metabolism. The fundamental validity of this finding is supported by our uncovering of a similar pattern of transcriptional alterations in *Pai-1*^+/−^ mice. Furthermore, in mice fed a HFHS diet, modulation of PAI-1 activity yields significant effects on the transcription of numerous critical regulators of lipid biosynthesis, including *Pcsk9*, *Srebp1a*, *Srebp1c*, *Srebp2*, and *HMG-CoA reductase*. These alterations directly lead to a reduction in plasma cholesterol, LDL-C, and HDL-C levels. Taken together, these data demonstrate that under normal conditions, modest alterations in plasma PAI-1 activity have a pervasive and physiologically significant impact on hepatic lipid metabolism. Furthermore, this newly identified regulatory role persists under conditions of metabolic stress brought on by feeding a HFHS diet.

Plasma PAI-1 levels are elevated in patients with NAFLD, and there is accumulating evidence that PAI-1 plays a causal role in the pathogenesis of this condition^16,17^. Recently, Lu et al.reported that a DNA methylation estimator of plasma PAI-1 levels (DNAm PAI-1) is a remarkably powerful predictor of hypertension, T2DM, NAFLD, and plasma lipid levels in humans^24^. While these and other studies have identified important statistical associations, a mechanism to explain the link between PAI-1 and NAFLD has not yet been previously reported. In our attempts to address this question in an unbiased manner, we systematically investigated the effects of TM5614 on hepatic gene expression, and in the process uncovered a novel function of PAI-1 in the development of dyslipidemia. To our knowledge, this is the first study that mechanistically associates PAI-1 with hyperlipidemia, and its remarkable juxtaposition in metabolic control regulating PCSK9.

The RNA-seq dataset presented here revealed a role for PAI-1 in the expression of numerous genes responsible for lipid metabolism. These exploratory findings were confirmed by selective qRT-PCR analysis indicating that PAI-1 inhibition reduces hepatic *Pcsk9* expression, as well as numerous hepatocellular enzymatic components that regulate de novo cholesterol synthesis and fatty acid metabolism. PCSK9 is broadly recognized as an important regulator in lipid metabolism, largely through its effects in promoting the degradation of LDL receptors, thus impairing receptor-mediated LDL-C clearance^25^. In clinical practice, PCSK9 is a validated target in lipid-lowering therapy. The administration of monoclonal antibodies blocking the binding of PCSK9 to the LDLR yields ~60% reduction in LDL-C levels of patients suffering from hypercholesterolemia^26^. The effects of PAI-1 inhibition on the hepatic expression of *Pcsk9* translated to a decrease in plasma PCSK9 concentrations in mice and a resultant significant reduction in serum cholesterol. This same dataset revealed a novel and robust effect of PAI-1 inhibition in increasing the expression of *Fgf21*. While PCSK9 suppression was consistently observed irrespective of diet and method of reducing PAI-1 activity (pharmacologic or genetic), the effects of TM5614 on hepatic *Fgf21* expression were only observed in healthy mice on a standard chow diet. While the metabolic effects and potential benefits of FGF21 are numerous, the beneficial effects of PAI-1 inhibition on lipid metabolism do not appear to involve FGF21.

In order to gain some insight into the potential utility of PAI-1 inhibition in patients with the metabolic syndrome, we studied the effects of TM5614 in a diet-induced dyslipidemia model. Oral administration of TM5614 effectively reduced hepatic *Pcsk9* mRNA by over 80% with a corresponding 55% decrease in plasma PCSK9 levels. These effects were accompanied by a significant reduction in plasma HDL-C and LDL-C. The observed decrease in both HDL-C and LDL-C reported here is consistent with prior studies in mice using monoclonal antibodies that block PCSK9 activity^27^. Importantly, other genes involved in lipid synthesis and metabolism were also downregulated in this experimental model of diet-induced dyslipidemia, including *Srebp1/2* and *HMG-CoA reductase*. These changes are likely secondary to the increased uptake of intracellular cholesterol caused by the loss of PCSK9^28^. However, a direct role for PAI-1 on these core lipid synthesis genes cannot be excluded. In contrast, while PAI-1 inhibition had a significant effect in reducing LDL-C in this model, it was not accompanied by the anticipated augmentation of LDLR protein in hepatic lysates^29^**.** The apparent lack of change in LDLR levels may reflect numerous competing effects present in the model, including a partial reduction in circulating PCSK9 levels, cellular heterogeneity in responses, the restoration of receptor density over time, or artifacts of experimental design, including duration of therapy and the use of whole cell extracts as opposed to purified membranes.

The experimental evidence presented here clearly indicates that PAI-1 regulates lipid metabolism in mice. There is a paucity of information on the relationship between PAI-1 and PCSK9 in the clinical literature. Importantly, we did find that genetic deficiency of PAI-1 is associated with a reduction in hepatic *Pcsk9* expression in mice and in human plasma PCSK9 levels. While this relationship is significant, it probably underestimates the effects of PAI-1 on PCSK9 levels. Multiple factors likely contribute to the relatively narrow range [19 ± 14 ng/ml (mean ± SD)] of plasma PAI-1 levels in the unaffected Amish individuals reported in this study, including lifestyle, diet and fitness, and these may have blunted the effects on plasma PCSK9. We also uncovered the presence of a relatively robust correlation between plasma levels of PAI-1 and PCSK9 in patients that do not have genetic PAI-1 deficiency. Interestingly, PAI-1 levels also decreased dramatically in hypercholesterolemic patients who were treated with monoclonal antibodies that inhibit the function but raise the levels of plasma PCSK9. This newly recognized relationship indicates that pharmacologic inhibition of PCSK9 in hypercholesterolemic individuals not only reduces LDL-C but may also provide a secondary cardiovascular benefit by concomitantly reducing plasma PAI-1. In aggregate, these findings strongly suggest the presence of a reciprocal regulatory loop between PAI-1 and PCSK9.

While the effects of PAI-1 on hepatic lipid metabolism are extensive and likely important, the molecular mechanisms that explain these novel effects are incompletely understood. The aggregate effects of PAI-1 on hepatic gene expression likely involve a complex physiological calculus that includes: (1) the direct binding of PAI-1 binding to LRP and effects of Jak/STAT signaling^30,31^; (2) PAI-1 inhibition of the uPA-dependent activation of proHGF and subsequent alterations in the binding of active HGF to hepatic cMET receptors^32^; and (3) augmented transcription of FGF21 and increased hepatic signaling through the heterodimeric FGFR/β-Klotho receptor^33–36^. Other mechanisms may be related to the effects of PAI-1 on the furin-dependent activation of the insulin receptor^37^.

**Figure 6:**
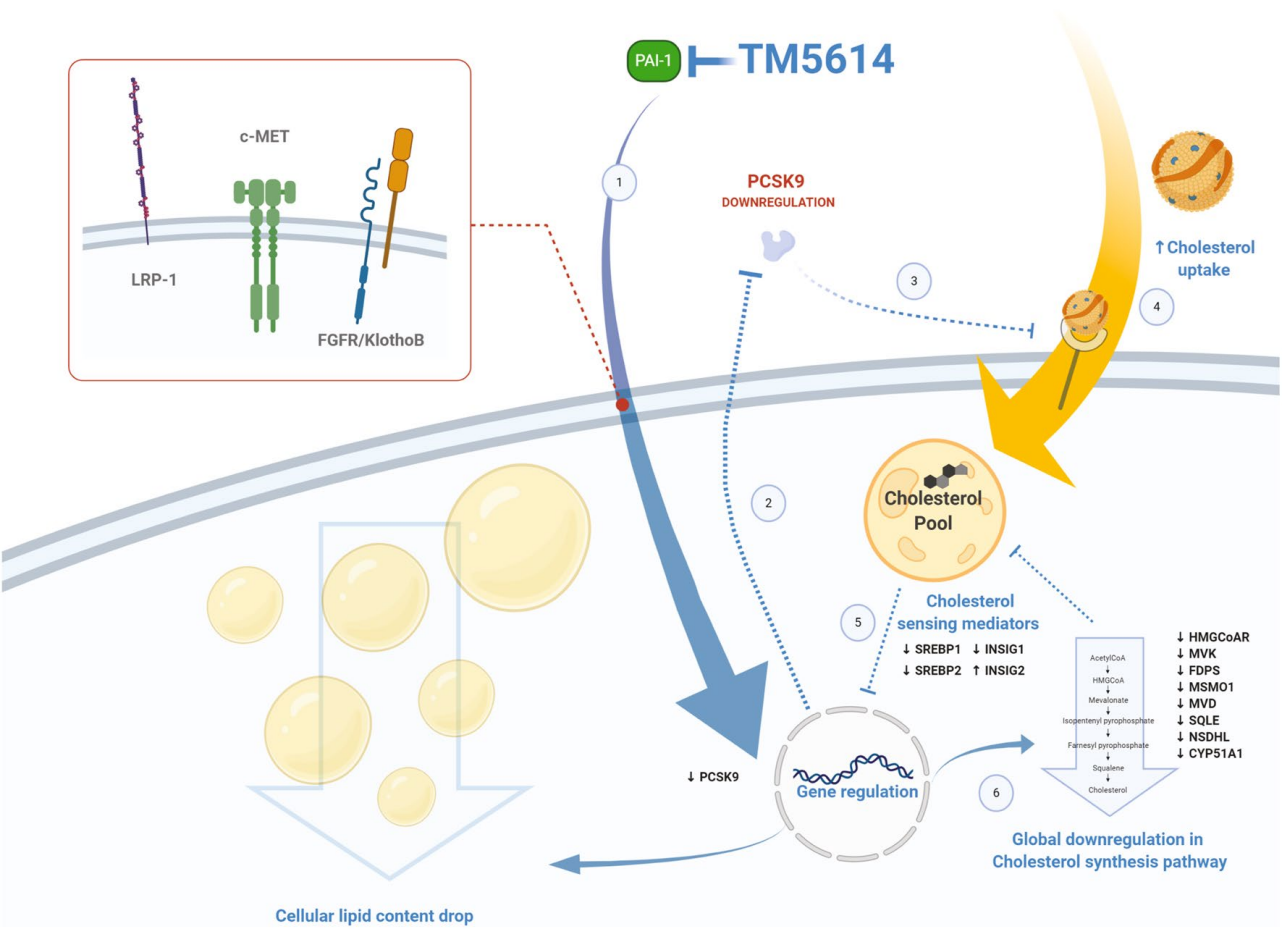
PAI-1 inhibition triggers a lipid-lowering effect mediated by PCSK9 downregulation. As TM5614 inhibits PAI-1 activity, hepatic PCSK9 is downregulated at the transcriptional level (1). Consequently, plasma levels of PCSK9 decrease (2). Lower plasma PCSK9 levels exert a lower impact on catabolism of the LDL receptor (3), enabling the receptor to clear larger amounts of LDL-C from the circulation (4). The increase in intracellular cholesterol triggers the translocation of cholesterol sensing factors to the nucleus (5) to downregulate the transcription of genes involved in lipid metabolism (6). LRP-1, c-MET or FGFR/KlothoB are potential mediators for the observed effect. Animation created with https://biorender.com/.

In conclusion, we have shown that pharmacological inhibition of PAI-1 prevents hepatic steatosis and reduces serum cholesterol levels through a mechanism that involves reduced PCSK9 synthesis (Fig. 6). Given that PAI-1 levels are consistently elevated in patients with the metabolic syndrome, our finding of a direct role for PAI-1 in the regulation of lipid metabolism provides further support to the central role of PAI-1 in the cardiovascular complications of the metabolic syndrome, and in the molecular pathogenesis of the metabolic syndrome itself. We anticipate that novel therapeutic agents that either reduce the synthesis or block the function of PAI-1 will be of value in the prevention and treatment of the metabolic syndrome and its vascular consequences.

## Materials and methods

### Materials

TM5614 (FW = 446.43 g/mol). A 20 mM stock solution was prepared in DMSO and stored at − 20 °C. In cell culture, TM5614 was added to a final concentration of 10 µM with DMSO < 0.5%. For animal feeding experiments, dry TM5614 was mixed into the powdered mouse food which were then formed into pellets given at a dose of 20 mg/kg/day.

### Human cohorts

Plasma samples from members of the Bern Amish Kindred who harbor a frameshift mutation in *SERPINE1* (*SERPINE1*^+*/*+^ (n = 17, 7 males) and *SERPINE1*^+*/*−^ (n = 16, 8 males) were assessed to quantify PCSK9 plasma levels. This study was previously approved by both the Northwestern University and the Indiana Hemophilia and Thrombosis Center Institutional Review Board as previously described^19^. All methods were performed in accordance with the relevant guidelines and regulations. Informed consent was obtained from all subjects.

Data was analyzed from a cohort of patients with heart failure with preserved ejection fraction (HFpEF). Patients were enrolled in this prospective observational study from the outpatient HFpEF clinic (for HFpEF patients) and from the outpatient general cardiology clinic or cardiac catheterization laboratory (elective cases) for the control patients. Control patients were deemed eligible if they had 1 or more risk factors for HFpEF (e.g., hypertension, obesity, diabetes, chronic kidney disease) and only if there was no history of heart failure (any type, including those with recovered LVEF). In this analysis, 248 unique plasma proteins (including PAI-1 and PCSK9) were quantified by a multiplex immunoassay (Olink, www.olink.com) using the commercially available panels cardiovascular disease II, IIII, and inflammation. This study was approved by the Northwestern University Institutional Review Board.

Plasma samples from hypercholesterolemic patients before and while on a PCSK9 inhibitory therapy (Alirocumab, 75 mg every 2 weeks, or Evolocumab, 140 mg every 2 weeks) were obtained from the OHSU Center for Preventive Cardiology Registry and Biorepository, approved by OHSU Institutional Review Board (17,329).

### Animal models

Wild-type C57BL/6 J male mice (Jackson Laboratories, Bar Harbor, ME) were fed a standard chow (SC) diet and underwent 14/10-h light/dark cycling with free access to food and water. Animal protocols were approved by the Northwestern University Institutional Animal Care and Use Committee (IACUC). A first cohort of 20-week-old mice was used to evaluate the acute effect of inhibiting PAI-1 with TM5614. Half of the mice were fed SC and the other half SC supplemented with TM5614 at a dose of 20 mg/kg/day for 10 days. A second cohort of 6-week-old mice was used to evaluate the long-term effects of inhibiting PAI-1 with TM5614 in mice fed a high-fat, high-cholesterol, high-sugar diet (HFHS). The HFHS diet is composed of 40% energy as fat (milk fat, 12% saturated) with 2% cholesterol (AIN-76 Western Diet, Test Diet) and drinking water supplemented with 42 g/l of 55% fructose/45% glucose by weight. Mice were fed HFHS for 4 months to induce obesity. After this period, half of the mice were treated with TM5614 (as described for the acute exposure) for a period of 2 months. A third cohort of mice *Pai-1*^+/−^ were generated by the breeding of *Pai-1*^−/−^ and WT mice (Jackson Laboratories, Bar Harbor, ME). These mice were maintained on SC.

At the end of these protocols, blood was collected by retro-orbital bleeding in citrated tubes and centrifuged to collect plasma and mice were euthanized by isoflurane followed by cervical dislocation. Mouse livers were flushed with ice-cold saline, a portion of the liver was put into RNA LATER (Cat# 76104, Qiagen) prior to RNA extraction. The remainder of the tissue was snap frozen in liquid nitrogen. All methods were performed in accordance with the relevant guidelines and regulations.

### RNA sequencing and analysis

RNA isolation and sequencing were performed as previously described^38^. Briefly, total RNA was extracted from murine livers using an RNA Easy mini kit (Cat#: 74104, Qiagen) and 1 µg of RNA were used for sequencing. RNA libraries were constructed using KAPA mRNA HyperPrep kits (Cat#kk8580, KAPA) according to the manufacturer’s instructions. Libraries were quantified using Bioanalyzer (Agilent) and sequenced on an Illumina NextSeq 500 instrument using 75 bp single-end reads. Sequenced reads were aligned to the mm10 reference mouse genome using STAR and differentially expressed RNAs were determined by analyzing with DESeq2 (FDR-adjusted * p* value < 0.05). STAR alignments and DESeq2 were performed though the RNA Express BaseSpace application (Illumina). DESeq 2 utilizes a Benjamini and Hochberg method to calculate the FDR. Morpheus (https://software.broadinstitute.org/morpheus/) was used to create a hierarchical clustering (heat map) was used to group genes on the basis of similar expression patterns over the samples and a volcano plot was generated to study the differentially expressed genes. The gene list was classified according to gene ontology biological processes. Metascape (http://metascape.org, June 2019) was used for ontology analysis.

### Gene expression

Total RNA was extracted from murine livers using the RNA Easy mini kit (Qiagen, Cat# 74104) and complementary DNA was synthesized from 1 µg of mRNA with the qScript cDNA Synthesis kit (Quantabio, Cat# 95048), in accordance with the manufacturer’s instructions. qRT-PCR was performed on 2 µL of cDNA using either SYBR-Green (Biorad, Cat# 1725270) or Taqman primer/probe mixes (Thermo Fisher Scientific). A table of primers can be found in the supplementary materials. The reactions were performed following the manufacturer’s instructions. GAPDH was employed as a housekeeping gene and fold changes in gene expression were calculated using the standard ∆∆Ct method.

### Plasma analysis

Total cholesterol was quantified in the murine plasma using an enzymatic determination (Pointe Scientific, Cat# C7510). The lipoprotein profile of the murine plasma samples was assessed running a fast-phase liquid chromatography (FPLC), in brief 100 µL of plasma (from one animal or a pool of several samples of the same conditions) were loaded to a Superose 6 column (GE HealthCare, Cat# 29-0915-96) at a flow rate of 0.5 mL/min in running buffer (0.15 M NaCl, 0.01 M Na2HPO4 and 1 mM EDTA). Fractions were collected every minute for 41 min and analyzed for cholesterol concentrations as described above. Murine plasma PCSK9 levels were determined using an ELISA kit (MBL International, Cat# CY-8078). Murine PAI-1 levels were quantified using an ELISA kit (Molecular Innovations, Cat# MPAIKT-TOT). Murine PAI-1 activity levels were quantified by ELISA (Molecular Innovations, Cat# MPAIKT). Human PAI-1 (Molecular Innovations, Cat# HPAIKT-TOT), PCSK9 levels (R&D Systems, Cat# DPC900), and FGF21 (R&D, DF2100) were quantified by ELISA.

### Hepatic protein quantifications

Hepatic proteins were extracted from liver tissue homogenized with lysis buffer (RIPA Buffer (Sigma-Aldrich, Cat# R0278) supplemented with 1X proteinase inhibitor (Thermo Fisher Scientific, Cat# A32963) and total protein content were performed as described^39^. mLDLR (R&D Systems, Cat# MLDLR0) protein levels were quantified from the total extracts (20 µg) using ELISA methodology following the manufacturers recommendation.

### In vitro cellular studies

Human hepatocellular carcinoma HepG2 (ATCC HB-8065) cell line was routinely grown as previously described^40^ at 37 °C in a 5% CO_2_ humidified atmosphere. 2.5 × 10^5^ cells were plated. The following day (day 2) media was changed to serum-free media. On day three cells could be treated with 10 µM TM5614 (or vehicle) for 48 h. On day five, cell culture media was collected and stored at − 20 °C until analysis. Cells were washed twice with ice-cold phosphate-buffered saline and scraped in 100 µL lysis buffer (same buffer as described for tissue protein extraction). Lysates were incubated on ice for 1 h (with mixing every 15 min) and spun at 14,000 × *g* at 4 °C for 20 min, supernatants were collected and quantified by a Lowry assay (DC Protein Assay, Cat# Bio-Rad, 5000111) then stored at − 20 °C until used. 20 µL of cell culture medium or 40 µg of total extracts were resolved in SDS-PAGE (4–12% or 12% Bis–Tris precast acrylamide gels, Thermo Fischer Scientific) and later transferred to nitrocellulose membranes (GE Healthcare, Cat# 10600003) and blot for human PCSK9 (using a rabbit anti-PCSK9 (1:1000 dilution, MBL International, Cat# CY-P1037) as a primary antibody and a goat anti-rabbit (1:15,000 dilution, LI-COR Biosciences, Cat# 925-32211) as the secondary antibody), LDLR (using a goat anti-LDLR (1:1000 dilution, R&D, Cat# AF2148) as the primary antibody and a donkey anti-goat (1:15,000 dilution, LI-COR Biosciences, Cat# 925-32214) as the secondary antibody), actin (using a mouse anti-actin (1:2000 dilution, Sigma-Aldrich, Cat# A5441) as the primary antibody and a goat anti-mouse (1:15,000 dilution, LI-COR Biosciences, Cat# 926-68070) as the secondary antibody).

### Glucose tolerance testing

Intraperitoneal glucose tolerance tests (IPGTT) were performed on mice fasted for 16 h. Glucose was injected by IP at 2 g/kg of body weight. Blood glucose was obtained from tail veins at multiple time points (0–120 min) and measured using a NovaMax Plus glucometer (Novacares).

### Hepatic triglyceride content

Hepatic triglyceride levels were measured using an Infinity spectrophotometric assay (Thermo Electron Corporation, Cat# TR22421) following lipid extraction using the Folch method^41^.

### Histologic analysis

Liver was fixed in 10% Phosphate Buffered Formalin (Newcomer Supply, Cat# 1090 N) and embedded in paraffin. Section were stained with hematoxylin and eosin (H&E) (Newcomer Supply, Cat# 1201 and 1082A).

### Statistical analysis

The RNA seq data was deposited to GEO accession numbers GSE140725 and GSE151597. Statistical analyses including unpaired t test, paired t test, linear regression, one-way ANOVA, and two-way ANOVA were performed in Prism version 8 (www.graphpad.com). * p* values of < 0.05 were considered statistically significant.

## Supplementary Information

Supplementary Information.

## Data Availability

RNA-seq data has been deposited in GEO SuperSeries accession numbers GSE140725 and GSE151597. All other data are available in the main text and supplementary materials. TM5614 was obtained via a material transfer agreement with RenaScience.
