# Supplementary material for: Role of PAI-1 in hepatic steatosis and dyslipidemia
Source: Sci Rep. 2021 Jan 11;11:430. doi: 10.1038/s41598-020-79948-x (PMC7801442; doi:10.1038/s41598-020-79948-x)
Supplement: Supplementary file 1 — Supplementary Information. [file 41598_2020_79948_MOESM1_ESM.docx]

**Role of PAI-1 in Hepatic Steatosis and Dyslipidemia**

**Authors:** Joshua A Levine^1^^, Carlota Oleaga^2^^, Mesut Eren^1^, Ansel P Amaral^1^, Meng Shang^1^, Elizabeth Lux^1^, Sadiya S Khan^1^, Sanjiv J Shah^1^, Yasuhiro Omura^1^, Nathalie Pamir^2^, Joshua Hay^2^, Grant Barish^1,3^, Toshio Miyata^4^, Hagai Tavori^2^, Sergio Fazio^2^, Douglas E Vaughan^1*^

^equal contribution

**Affiliations:**

^1^Department of Medicine, Northwestern University Feinberg School of Medicine, Chicago, IL, USA.

^2^Center for Preventive Cardiology, Knight Cardiovascular Institute, Oregon Health & Science University, Portland, OR, USA.

^3^Department of Medicine, Jesse Brown VA Medical Center, Chicago, IL, USA.

^4^Department of Molecular Medicine and Therapy, United Centers for Advanced Research and Translational Medicine, Tohoku University Graduate School of Medicine, Miyagi, Japan.

**Material Requests and correspondence to:**

Douglas E. Vaughan, MD

Northwestern University Feinberg School of Medicine

Arkes Pavilion, Suite 2330

676 N. St. Clair Street

Chicago, IL 60611-2927

Phone 312-926-9436

Fax 312-926-0239

E-mail: [d-vaughan@northwestern.edu](mailto:d-vaughan@northwestern.edu)

**Supplementary Materials**

**Supplemental Figures legends**

**Supplemental Table 1:** List of RT-PCR primers or TaqMan assay numbers

**Supplemental Figure 1. Shared transcriptional regulation induced by *Pai-1* heterozygosity and TM5614 treated mice.** Hepatic mRNA from two murine cohorts were compared. Cohort #1: 20-week-old wild-type C57BL/6J male mice fed SC, half were challenged with the acute exposure to a PAI-1 inhibitor, TM5614 given orally (20 mg/kg/day) for ten days. Cohort #2: 20-week-old wild-type and *Pai-1*^+/-^ C57BL/6J male mice fed SC were compared. A. Hierarchical clustering of expression changes seen by RNA-seq in Cohort #1. Heat map created using Morpheus (<https://software.broadinstitute.org/morpheus/>) B. Hierarchical clustering of expression changes seen by RNA-seq in cohort #2. Heat map created using Morpheus (<https://software.broadinstitute.org/morpheus/>) C. Venn diagram to highlight the shared genes differentially regulated in the two PAI-1 cohorts. * common gene between 2 cohorts

**Supplemental Figure 2. TM5614 reduces the HDL-cholesterol levels in mice fed chow diet.** 20-week-old wild-type C57BL/6J male mice fed chow were challenged with the acute exposure to a PAI-1 inhibitor, TM5614 given orally (20 mg/kg/day) for ten days. At the endpoint murine plasma was analyzed. A. Cholesterol content profile of the lipoprotein fractions resolved by FPLC from pooled samples (n=5 for control, n=5 for TM5614). B. Quantification of cholesterol in each lipoprotein fraction calculated using AUC from the FPLC profile.

**Supplemental Figure 3: *In vitro* characterization to TM5614 incubation.** HepG2 cells were incubated with TM5614 (10 µM) or vehicle (DMSO <0.5%) for 48 h. Cell conditioned media and extracts were resolved by SDS-PAGE and western blotted for PCSK9, LDLR and actin. A. PCSK9 levels. Image is representative of two independent experiments. B. Quantification of PCSK9 levels seen in panel A. C. LDLR levels. D. Quantification of LDLR levels seen in panel C. Image contains three independent experiments. Values are express as mean ± SEM (n=4), * p<0.05 and ** p<0.01, *** p<0.001.

**Supplemental Figure 4. TM5614 improves glucose tolerance and hepatic steatosis.** A. Initial weights of mice prior to TM5614 treatment. B. Percentage weight change during the period of TM5614 administration. C. Intraperitoneal glucose tolerance test. P value represents a 2 way ANOVA. D. Hematoxylin and Eosin staining of liver. E. Quantification of triglyceride content. Values are expressed as mean ± SEM (n=6 HFHS 7 HFHS + TM6514), ** p<0.01.

**Supplemental Figure 5. Changes induced in PCSK9 plasma levels.** A comparison between PCSK9 plasma levels quantified in the murine models. Values are expressed as mean ± SEM (n=6-7), ** p<0.01.

**Supplemental Figure 6. PCSK9 levels increase after administration of a PCSK9 monoclonal antibodies.** Human plasma PCSK9 levels from before and after administration with a monoclonal antibody against PCSK9. Values are express as mean ± SEM (n=28), p<0.0001.

**Supplemental Figure 7.** ***In vitro* characterization to TM5614 incubation.** Full image of western blot from A. Figure S3A cell extract. B. Figure S3A cell media. C. Figure S3C


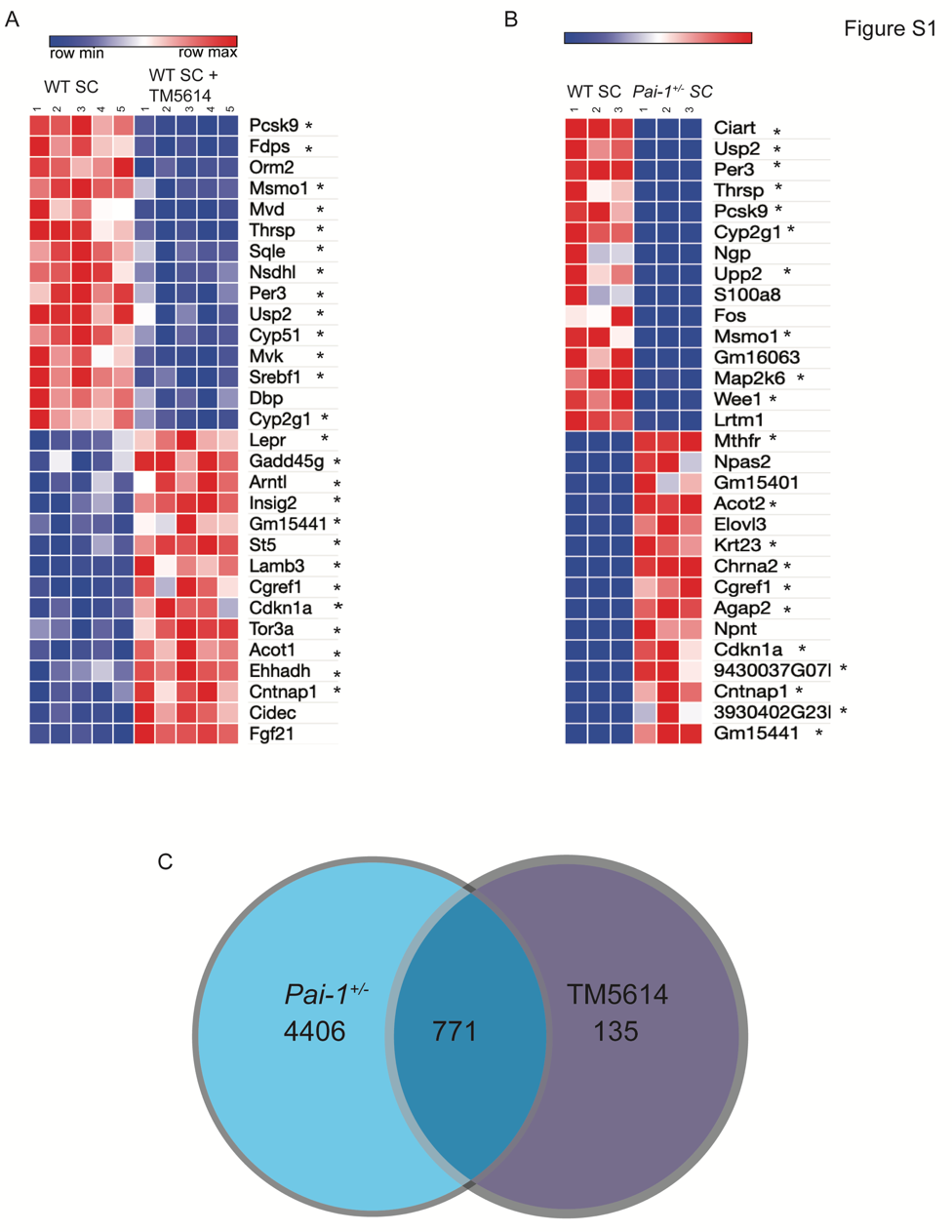


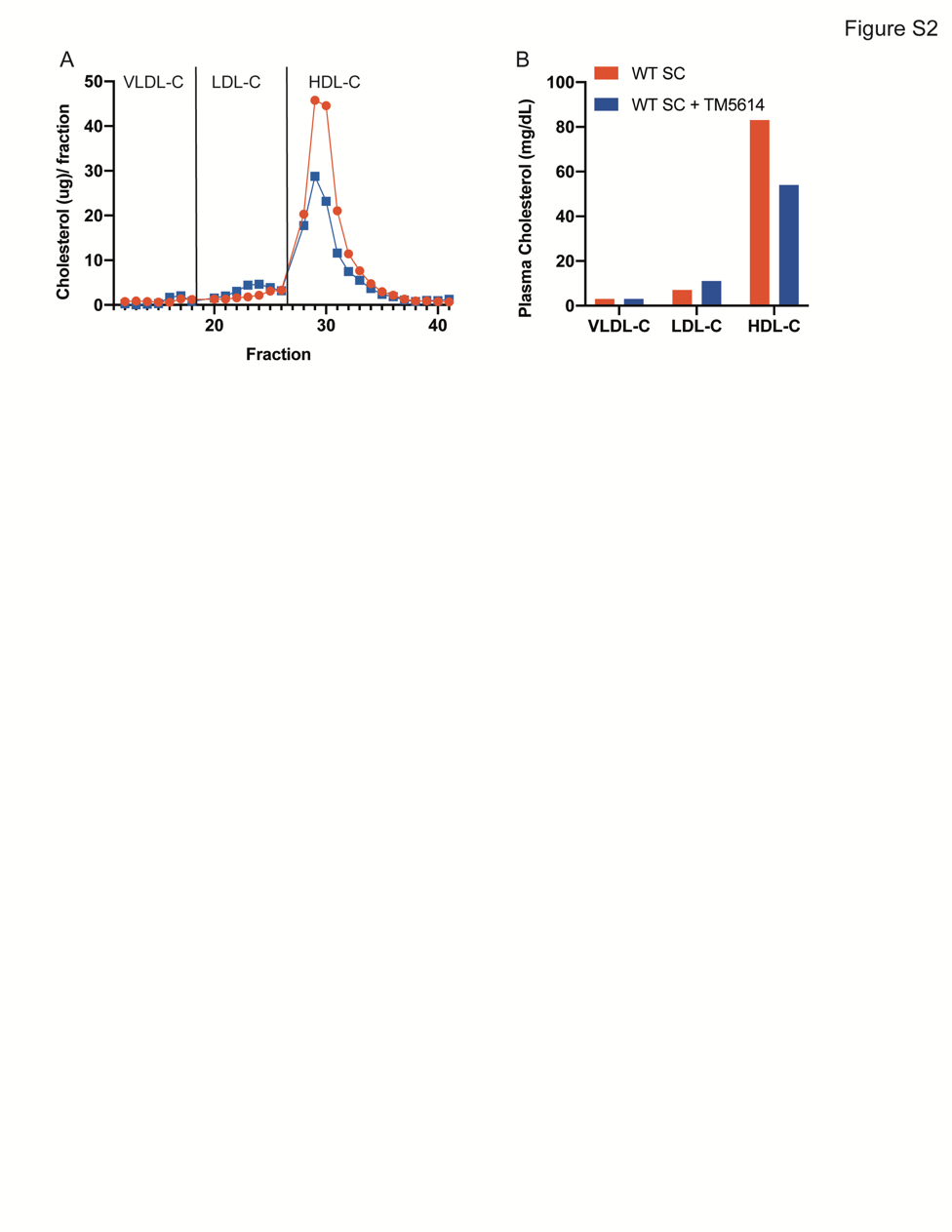


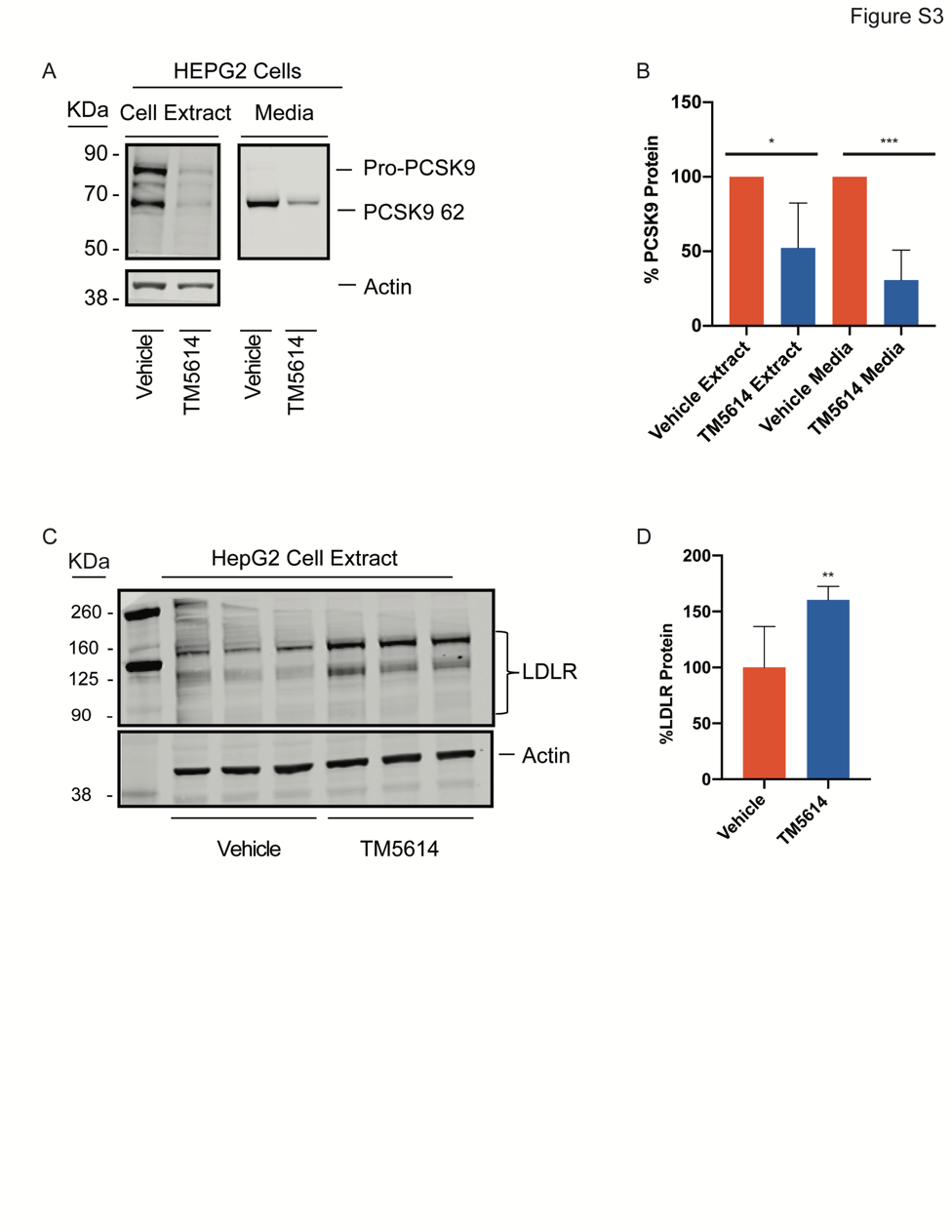


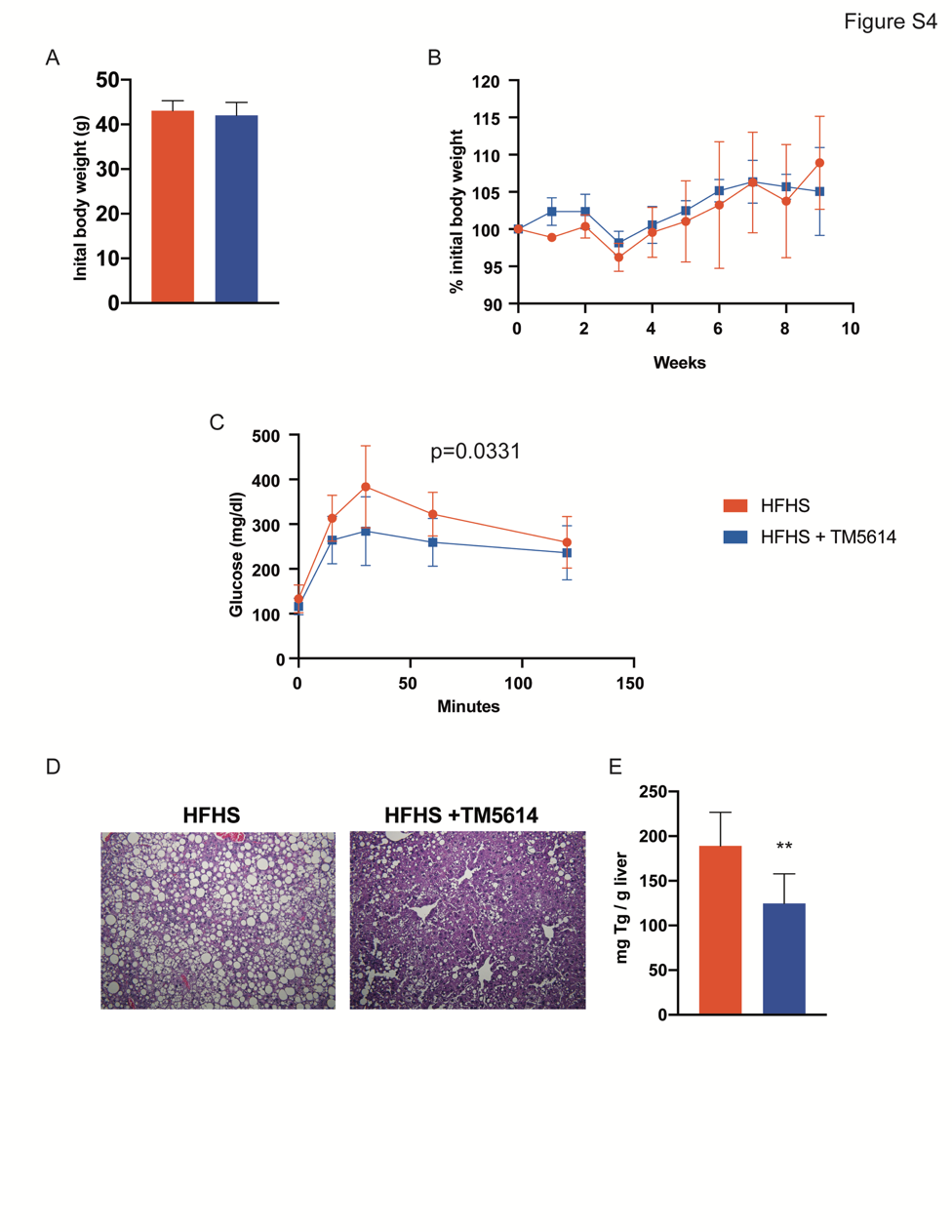


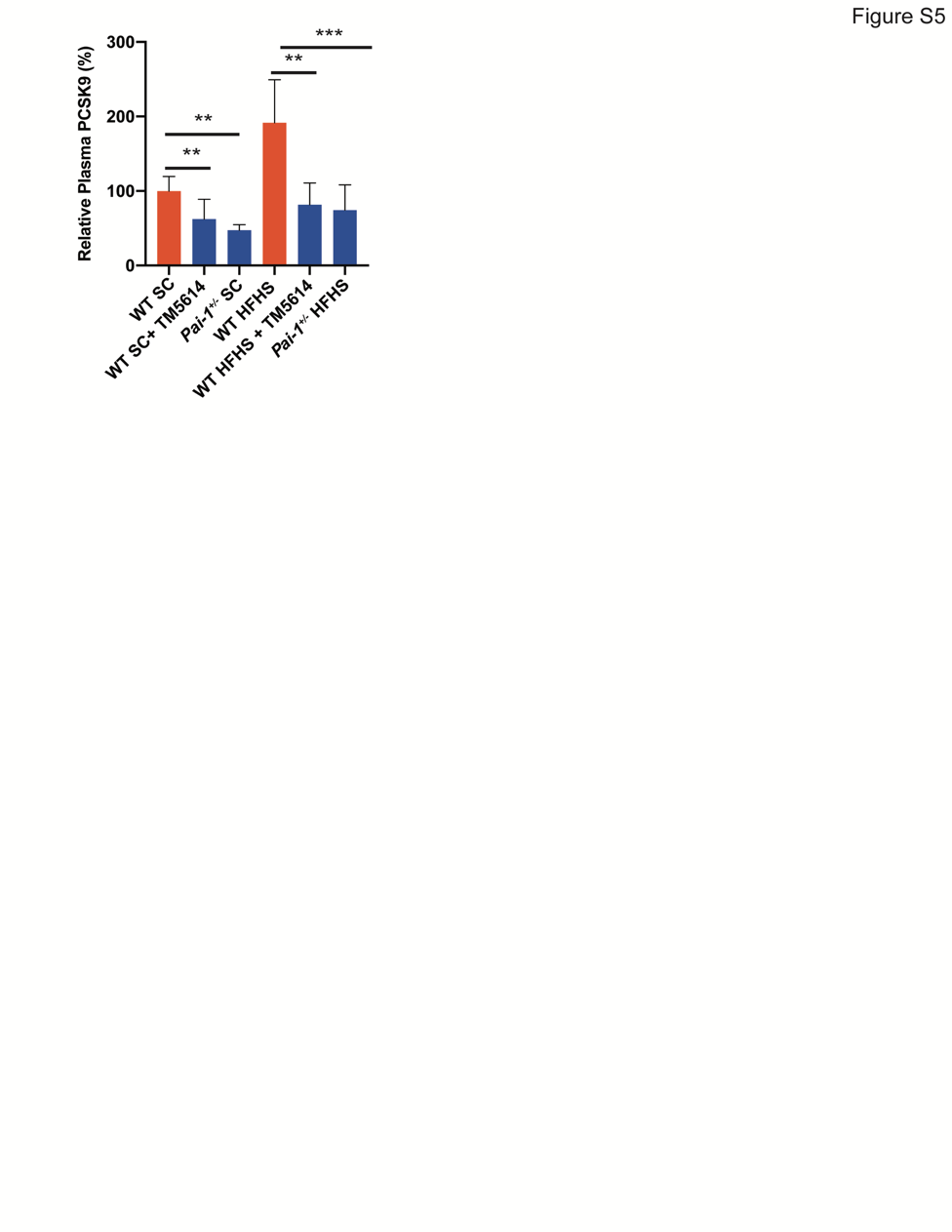


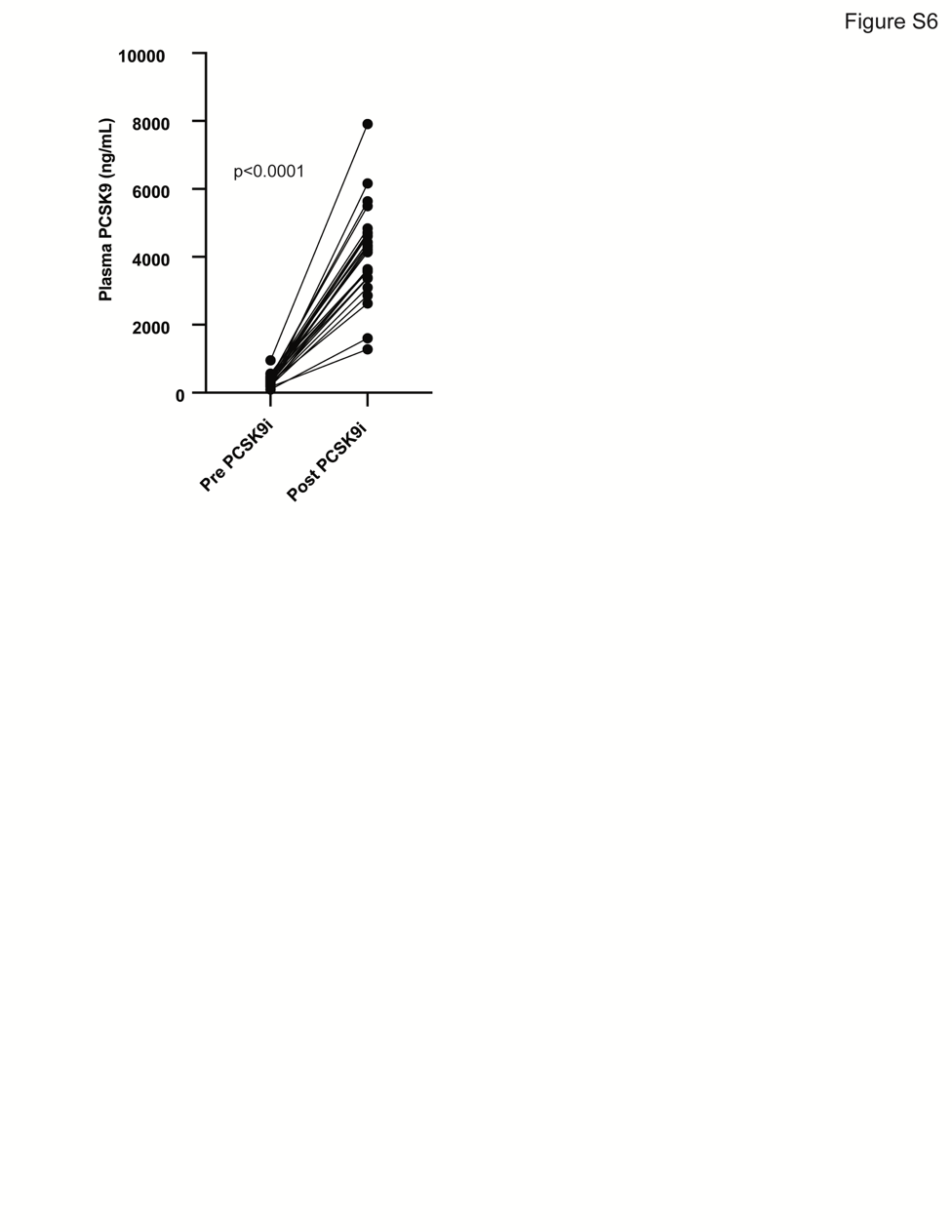


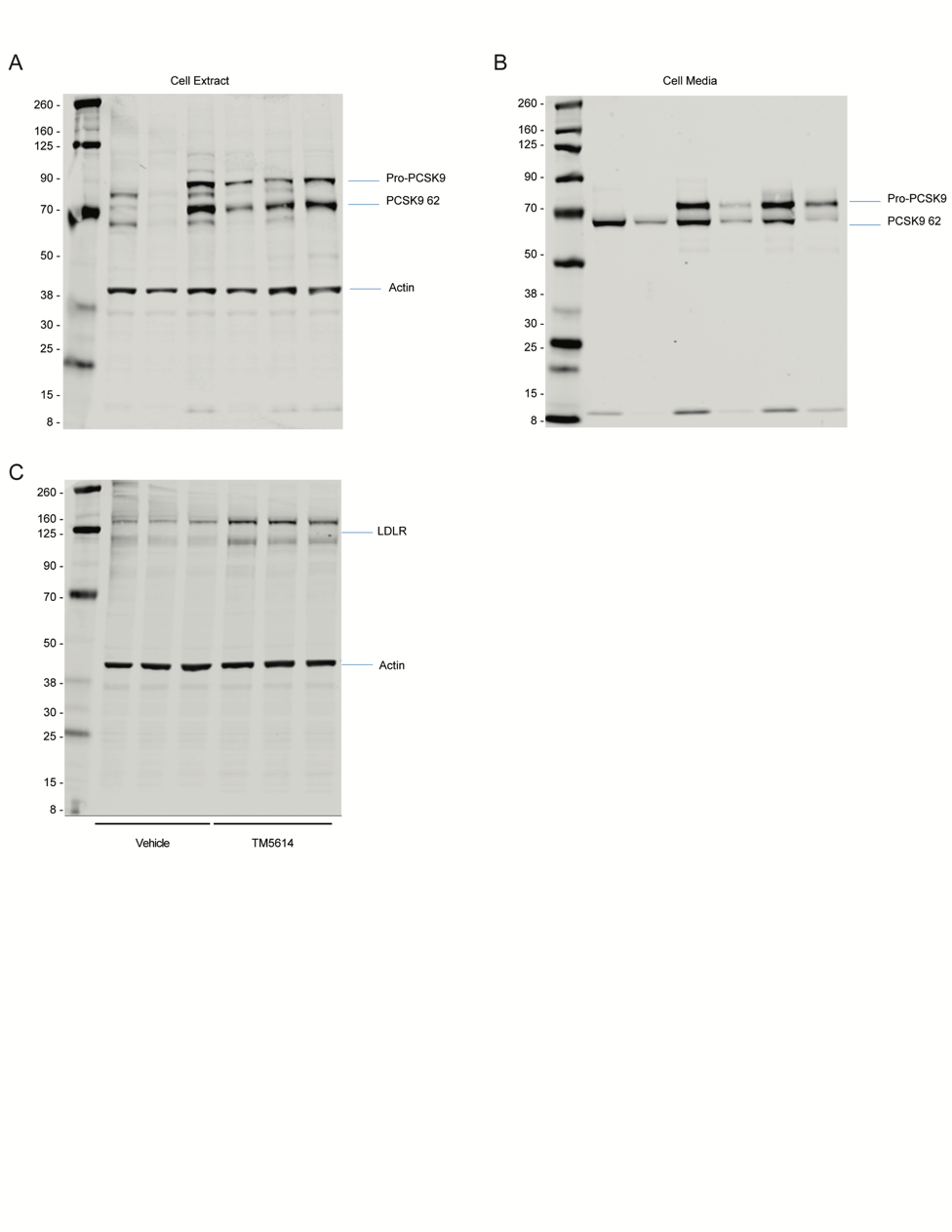


| **Gene** | **5’ Primer** | **3’ Primer** |
| --- | --- | --- |
| *Pcsk9* | TGGCTGCATGACATTGCTTCT | GCACTGGAGAACCACACAGG |
| *Srebp1a* | GCGCCATGGACGAGCTG | TTGGCACCTGGGCTGCT |
| *Srebp1c* | GGAGCCATGGATTGCACATT | GCTTCCAGAGAGGAGGCCAG |
| *Srebp Total* | GCTCCAGCTCATCAACAACCA | CAGGAAGGCTTCCAGAGAGGA |
| *Srebp2* | CCCTTGACTTCCTTGCTGCA | GCGTGAGTGTGGGCGAATC |
| *HMG CoA Reductase* | GATTCTGGCAGTCAGTGGGAA | GTTGTAGCCGCCTATGCTCC |
| *Ldlr* | Mm00440169_m1 |  |
| *Gapdh* | ATGTTCCAGTATGACTCCACTCACG | GAAGACACCAGTAGACTCCACGACA |
